# Efficacy and safety of through-the-scope twin clip for endoscopic closure of gastrointestinal defects

**DOI:** 10.3389/fmed.2026.1727989

**Published:** 2026-03-04

**Authors:** Chenyang Li, Yan Xu, Zhongrui Xu, Yujie Feng, Tao Wang, Fuxiu Huang, Ningning Zhang, Chuangxin Zhang, Sang Zhou, Shuling Li, Chao Chen

**Affiliations:** 1 Department of Gastroenterology, The First Medical Center of PLA General Hospital, Beijing, China; 2 Department of Gastroenterology, The Fourth Medical Center of PLA General Hospital, Beijing, China; 3 Department of Anesthesiology, The Fourth Medical Center of PLA General Hospital, Beijing, China; 4 Department of Cardiology, The PLA Rocket Force Characteristic Medical Center, Beijing, China

**Keywords:** defect closure, dual action tissue clip, endoscopic closure, endoscopic surgeries, through-the-scope twin clip

## Abstract

**Background and aims:**

The widespread adoption of endoscopic therapy has made the closure of gastrointestinal defects a critical limiting factor for its advancement. Recently, a novel device—the through-the-scope twin clip (TTS-TC)—has been introduced for endoscopic defect closure. This study aimed to evaluate the safety and efficacy of TTS-TC.

**Methods:**

A retrospective observational study was conducted at a territory center, involving patients who underwent endoscopic closure using the TTS-TC from February 2023 to December 2023. The TTS-TC features an additional fixed support column centrally positioned between the bilateral metal clips, enabling independent operation. We collected data on patient demographics, lesion characteristics, endoscopic closure time, procedural outcomes, and post-procedural outcomes, including adverse events.

**Results:**

Among the 36 recruited patients (22 male, 14 female), the mean age was 61 ± 12 years. Most interventions were performed in the stomach (61.1%). Indications for clip application included hemostasis of acute ulcer bleeding (*n* = 2), closure of perforations (*n* = 6), and deep-wall lesions (*n* = 28). Successful TTS-TC placement was achieved in all 36 patients (36/36). The mean maximum lesion size was 3.5 ± 1.0 cm, with a mean procedural time of 3.5 ± 1.8 min. The mean postoperative hospital stay was 5.7 ± 3.0 days. Full-thickness resection was associated with more adverse events compared with the non-FTR group, including intraprocedural perforation, abdominal pain, and fever (*p* < 0.001). No clip-related adverse events were observed.

**Conclusion:**

The TTS-TC emerges as a promising tool for the closure of diverse gastrointestinal defects, offering a new dimension in endoscopic management.

## Introduction

Endoscopic therapy has advanced considerably, broadening its role in managing a wide range of gastrointestinal conditions. Techniques such as endoscopic mucosal resection (EMR), endoscopic submucosal dissection (ESD), and endoscopic full-thickness resection (EFTR) have become cornerstones for treating lesions from benign mucosal abnormalities to early-stage malignancies. These minimally invasive approaches offer significant advantages over traditional surgery by preserving organ integrity and expediting postoperative recovery (1). However, they have concurrently underscored the critical need for reliable mucosal defect closure, making prophylactic closure an essential component in advancing endoscopic surgery.

Various devices have been developed for endoscopic defect closure, including traditional through-the-scope clips (TTSC), endoscopic suturing systems, and over-the-scope clips (OTSC). The management of large or perforated defects has long posed a major challenge in endoscopic therapy, though recent advances in instrumentation now provide effective solutions. OTSC, historically the mainstay for such closures, has demonstrated proven safety and efficacy worldwide (2, 3). Nevertheless, its application entails certain limitations, notably the requirement for pre-procedural mounting onto the endoscope tip, which may necessitate additional instrument exchanges and prolonged operative times, thereby increasing the risk of complications such as postoperative peritonitis, particularly in cases of full-thickness resection or perforation.

The continuous refinement of endoscopic instruments has led to a gradual resolution of the challenges associated with defect closure. Among these advancements is the introduction of the through-the-scope twin clip (TTS-TC), a novel device that has garnered widespread interest within the endoscopic community (4). This study aimed to rigorously assess the TTS-TC, evaluating its safety and efficacy in the context of endoscopic closure for a variety of gastrointestinal defects.

## Methods

### Setting and participants

From February to December 2023, 36 patients underwent TTS-TC placement consecutively due to ulcer bleeding or iatrogenic bleeding, deep wall lesions, or perforations in the stomach, colon, or rectum. Informed consent was secured from all participants prior to undergoing endoscopic interventions, in accordance with ethical standards. All procedures were performed by two highly experienced endoscopists. The procedures were performed on conscious patients or those under deep sedation with the support of an anesthesiologist. This study was approved by the Biomedical Research Ethics Committee of the Fourth Medical Center of Chinese PLA General Hospital.

### The TTS-TC system

**Figure 1:**
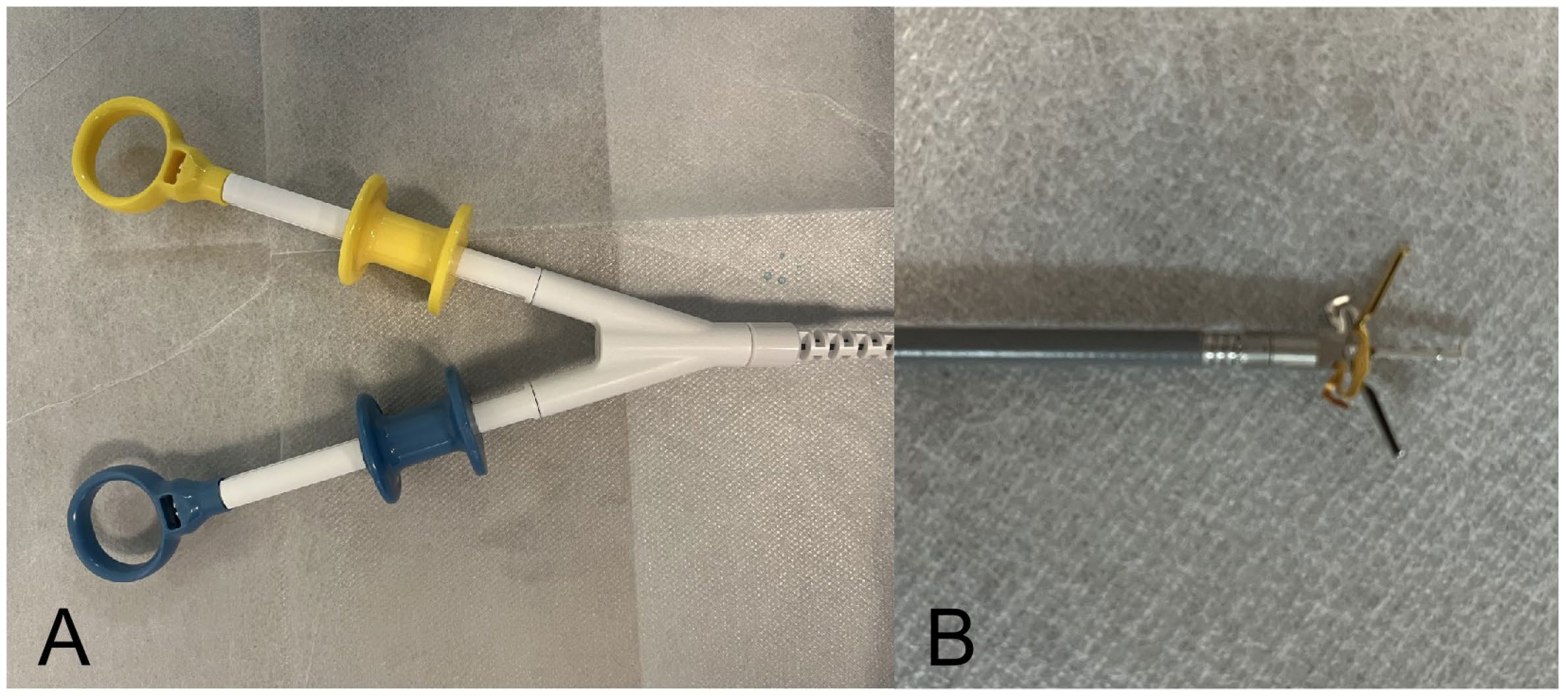
The through-the-scope twin clip. The clips are controlled independently by two handles. The gold clip is controlled by the gold handle and the blue clip is controlled by the blue handle. **(A)** Operation handles. **(B)** Both clips opened.

The main endoscopic instruments in this study were a gastroscope (Q260J; Olympus, Tokyo, Japan), IT knife (Olympus), conventional TTSC (ROCC-D-26-195-C and ROCC-F-26-195-C; Micro-Tech Co, Ltd., Nanjing, China), and the novel TTS-TC (Micro-Tech Co, Ltd., Nanjing, China). The TTS-TC features a central support column that enables independent operation of its bilateral clips, with a maximum outer diameter of 2.9 mm, an opening angle of up to 60 degrees, and an opening size of 1.0 cm (Figure 1).

### Operation procedure

**Figure 2:**
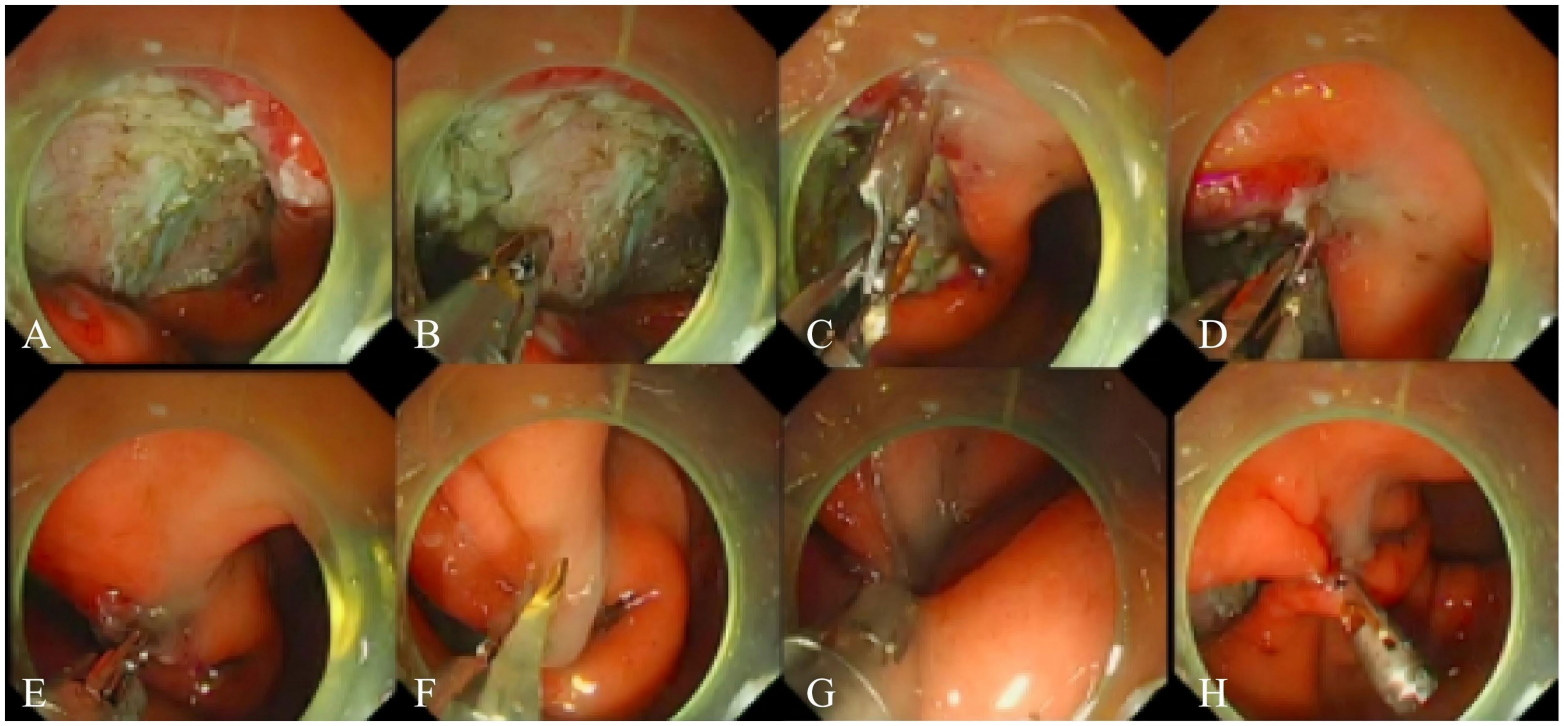
Operation steps using TTS-TC for defect closure. **(A)** A defect was located in the gastric antrum. **(B)** The TTS-TC was inserted into the gastric cavity through the endoscopic working channel. **(C)** The clip on one side of the TTS-TC was opened. **(D)** The clip clamped the mucosa on one side of the defect. **(E)** The clamped mucosa was pulled towards the opposite of the defect. **(F)** The clip on the other side of the TTS-TC was opened. **(G)** The clip clamped the mucosa on the other side of the defect. **(H)** The clips on both sides of the TTS-TC were locked and released, resulting in the large defect becoming several small defects.

The TTS-TC was introduced into the gastrointestinal tract via the endoscope’s working channel (Figure 2; Supplementary Video 1). One side of the TTS-TC was opened to clamp the edge of the defect. The mucosa, once clamped, was carefully repositioned to approximate the opposite edge of the defect. The clip on the other side of the TTS-TC was then opened to clamp the opposite edge. Once both edges of the defect were secured, the clips were locked. The TTS-TC was then released and retained *in vivo*. Additional traditional mental clips were deployed to achieve complete closure.

### Data collection

Demographic information about patients, lesion characteristics, endoscopic closure time, procedural outcomes, and post-procedural outcomes, including adverse events, were extracted from electronic medical records. Follow-up assessments were conducted at the discretion of the endoscopist, based on the perceived necessity. Otherwise, routine follow-up intervals were performed based on individual clinical pathology and procedure.

### Outcomes

Technical success was defined as the complete closure of the defect. Clinical success was defined as the resolution of the issue, such as the absence of the need for surgery or further endoscopic intervention. The operating time using TTS-TCs was defined as the duration between clip insertion and release. The total operating time for endoscopic closure was defined as the duration between the end of dissection or hemostasis and complete closure of the defect. Full-thickness resection was defined as the presence of all gastrointestinal wall segments in the resected specimen. Adverse events were graded according to a standardized adverse events classification system for GI endoscopy, including intraprocedural perforation, abdominal pain, delayed bleeding, fever, and so on (5).

### Statistical analysis

Descriptive statistics were calculated for all demographic, imaging, and clinical variables and were reported as mean ± SD, or as a proportion. Univariate analysis was performed by using the chi-square test the Fisher exact test for categorical variables and the t-test for continuous variables as required. All statistical analysis was conducted by using SPSS version 22.0 (IBM, Armonk, NY). A *p* <0.05 was considered significant.

## Results

**Figure 3:**
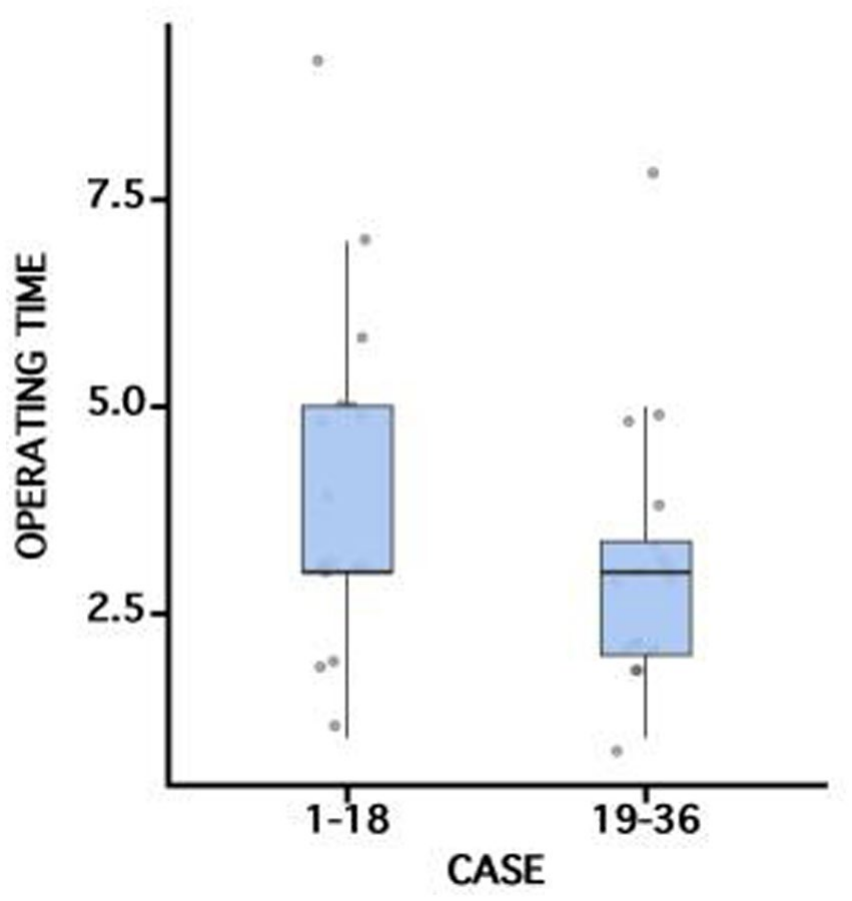
Comparison of operating time using TTS-TCs between different cases.

Out of the 36 patients recruited, 22 were male and 14 were female (Table 1; Supplementary Table 1). The mean age of the patients was 61 ± 12 years. The majority of the interventions were performed in the stomach (61.1%), followed by the colon (25.0%) and the rectum (13.9%). The indications for clip application were hemostasis of acute ulcer bleeding (*n* = 2), closure of perforations (*n* = 6), and deep-wall lesions (*n* = 28). The average number of clips used was 1.5 ± 0.8 (Table 2). The mean maximum diameter of the lesions was 3.5 ± 1.0 cm. The TTS-TC was successfully placed in all 36 patients (100% technical success) with the clip correctly deployed at the site of the defect. The clinical success rate was 100%. The TTS-TC operating time was 3.0 (3.0–5.0) min for the first 18 patients and 3.0 (2.0–3.4) min for the subsequent 18 patients; the difference between groups was not statistically significant (*p* > 0.05) (Figure 3). Final pathology showed full-thickness resection in 6 patients. All patients were hospitalized for observation following TTS-TC placement. The median hospital stay was 5.7 ± 3.0 days, which depended mainly on comorbidities. There was no difference observed between locations in terms of the number of clips used, operation time using TTS-TCs, or hospital stay after TTS-TC placement (Table 3).

**Table 1:** Demographic and clinical characteristics of patients in the study.

Characteristics	Values
Age (years)	61 ± 12
Female gender	14(38.9%)
Location	
Stomach	22(61.1%)
Colon	9(25.0%)
Rectum	5(13.9%)
Indication	
SEL	16(44.4%)
Early cancer	16(44.4%)
Perforation	6(16.7%)
Bleeding	2(5.6%)
Procedure	
Wound closure after ESD	27(75.0%)
Wound closure after ESE/EFTR	6(16.7%)
Perforation closure	6(16.7%)
Hemostasis	2(5.6%)

Data are n(%) or mean (±SD). SEL, Subepithelial Lesions; ESD, Endoscopic Submucosal Dissection; ESE, Endoscopic Submucosal Excavation; EFTR, Endoscopic Full-thickness Resection.

**Table 2:** Clinical outcomes.

Outcomes	Values
Lesion size (cm)	3.5 ± 1.0
Number of TTS-TCs	1.5 ± 0.8
Operating time using TTS-TCs (min)	3.5 ± 1.8
Total operating time for endoscopic closure (min)	19 ± 11
Technical success	36(100%)
Clinical success	36(100%)
Stay in hospital after TTS-TC placement (d)	5.7 ± 3.0
Adverse events	10(27.8%)
Intraprocedural perforation	6(16.7%)
Abdominal pain	4(11.1%)
Delayed bleeding	2(5.6%)
Fever	1(2.8%)

Data are n(%) or mean (±SD).

**Table 3:** Comparisons between different subgroups.

Characteristics	Location			*p*-value	FTR		*p*-value
	Stomach	Colon	Rectum		Yes	No	
	*n* = 22	*n* = 9	*n* = 5		*n* = 6	*n* = 30	
Number of TTS-TCs	1.6(0.9)	1.3(0.7)	1.2(0.5)	0.535	1.3(0.5)	1.5(0.9)	0.539
Operating time using TTS-TCs (min)	3.3(1.8)	4.0(2.4)	3.7(1.3)	0.645	3.8(2.9)	3.5(1.6)	0.782
Total operating time for endoscopic closure (min)	19(10)	18(12)	20(14)	0.956	26(16)	18(10)	0.29
Stay in hospital after TTS-TC placement (d)	6.4(3.1)	4.7(2.9)	4.4(1.1)	0.192	7.8(2.6)	5.3(2.9)	0.061
Adverse events	7(31.8%)	2(22.2%)	1(20.0%)	0.877	6(100%)	4(13.3%)	**<0.001**

Data are n(%) or mean (±SD). FTR, Full-thickness Resection.

Bold values indicate statistical significance.

Adverse events occurred in 10 patients. Full-thickness resection resulted in more adverse events compared with the non-FTR group (Table 3), such as intraprocedural perforation, abdominal pain, and fever (100%, *p* < 0.001). Four patients experienced adverse events after undergoing ESD/ESE, including mild abdominal pain and bloody stools, which improved after conservative treatment. The occurrence of adverse events is believed to be related to the depth of the defect and whether or not the entire layer is removed. No procedure-related complications directly associated with the TTS-TC were encountered.

## Discussion

Endoscopic resection technology has expanded markedly in clinical practice for diverse gastrointestinal pathologies, including peptic ulcer bleeding, early-stage neoplasms, and subepithelial lesions. As the scope of interventions has broadened, so have the challenges of closing iatrogenic or spontaneous defects. While conventional through-the-scope clips (TTSC) have traditionally served this purpose, the evolution toward more extensive procedures and complex defects has intensified the demand for innovative solutions. Current options include over-the-scope clips (OTSC), purse-string devices combining clips with loops or sutures, dedicated endoscopic suturing systems (OverStitch, X-Tack), and fibrin sealant application (6–12). Each modality presents distinct advantages and limitations. Achieving rapid, effective, and economical closure of gastrointestinal defects remains a pivotal challenge in advancing endoscopic surgery.

This study delves into the application of TTS-TC in endoscopic therapy, a device initially developed and implemented by Prof. Zhang’s team (4). The TTS-TC, also recognized as the dual action tissue (DAT) clip, has exhibited exemplary performance in preclinical and clinical settings, effectively sealing defects and minimizing complications (4, 13–16). Compared with TTSCs, the TTS-TC is distinguished by an additional fixed support column located centrally between bilateral metal clips, which facilitates independent clip operation. The bilateral clips are controlled independently via two color-coded handles and achieve a maximum opening size of 1.0 cm, making the device suitable for effective closure of defects less than 5.0 cm in diameter. The study showcases the successful deployment of TTS-TC in both endoscopic resection of gastrointestinal lesions and the management of gastric ulcer bleeding, achieving both technical and clinical success rates of 100%, comparable to those of OTSC (2, 3). The TTS-TC was utilized in treating lesions between 2.0 and 5.0 cm in diameter, with the largest cases involving 22 gastric and 14 colorectal instances. The average operation time for TTS-TC was 3.5 ± 1.8 min, with an average closure time of 19 ± 11 min. With increasing operator experience, TTS-TC operating time tended to decrease. However, our data showed no statistically significant difference between the first and second patient groups, likely attributable to heterogeneity in lesion locations and the relatively small sample size. We plan to investigate the TTS-TC learning curve in future studies. The use of TTS-TC also correlated with reduced operation times and exposure to defects, leading to enhanced patient outcomes. Despite its promising performance, the TTS-TC’s rotation flexibility is less than that of the newer generation TTSCs, suggesting areas for future refinement.

In this study, six instances involving full-thickness resection and perforation were successfully managed with the through-the-scope twin clip (TTS-TC), achieving effective closure. Postoperatively, these cases reported transient mild abdominal discomfort and low-grade fever, indicative of a controlled peritoneal inflammatory response. These symptoms were promptly resolved with conservative management. A statistical analysis revealed a significant association between the occurrence of adverse events and the depth of the defect, as well as the extent of tissue excision, including full-thickness resections (*p* < 0.001). The findings underscore the TTS-TC’s efficacy in managing sizable defects, with the capacity to seal lesions up to 5.0 cm in diameter, boasting both technical and clinical success rates of 100%. The deployment of the TTS-TC through the endoscope channel enhances operative efficiency and minimizes the risk of postoperative complications. This technique offers a distinct advantage over the over-the-scope clip (OTSC) and conventional through-the-scope clips (TTSC) in the context of defect closure. The study’s results suggest that the TTS-TC is a valuable addition to the endoscopist’s toolkit, offering a safe and effective alternative for endoscopic defect management. However, to fully elucidate the comparative benefits and optimal applications of various clip systems in clinical practice, further well-designed, controlled trials are warranted. These studies will be instrumental in refining the guidelines for the use of these innovative devices, thereby enhancing the overall safety and efficacy of endoscopic procedures.

The study acknowledges several limitations, including its retrospective design and the inherent selection bias associated with a single-center study. The modest sample size also constrains the generalizability of the findings, warranting larger-scale studies to better understand the closure effects across various defect types. Moreover, additional preclinical research is warranted to ascertain the anatomical changes post-TTS-TC closure in full-thickness resection or perforated patients.

## Conclusion

In summary, the TTS-TC has demonstrated robust reliability and efficacy as an endoscopic tool for managing an array of gastrointestinal defects. Its application has been effective in addressing issues arising from bleeding, perforations, and defects after endoscopic resections. The TTS-TC has achieved a high rate of closure success and has been associated with a minimal occurrence of adverse events, suggesting a favorable safety profile. Nevertheless, further research is encouraged to validate these preliminary findings and explore the broader application of TTS-TC in diverse clinical scenarios.

## Data Availability

The original contributions presented in the study are included in the article/Supplementary material, further inquiries can be directed to the corresponding author.

## Supplementary material

SUPPLEMENTARY TABLE 1 Demographic and clinical characteristics of patients in the study.

SUPPLEMENTARY VIDEO 1 Operation steps using TTS-TC for defect closure.
